# Epstein-Barr virus infection as potential indicator of the occurrence and clinical presentation of systemic lupus erythematosus

**DOI:** 10.3389/fimmu.2023.1307589

**Published:** 2023-12-07

**Authors:** Ana Banko, Andja Cirkovic, Rada Miskovic, Ivica Jeremic, Milka Grk, Milica Basaric, Ivana Lazarevic, Sanvila Raskovic, Aleksa Despotovic, Danijela Miljanovic

**Affiliations:** ^1^ Institute of Microbiology and Immunology, Faculty of Medicine, University of Belgrade, Belgrade, Serbia; ^2^ Institute for Medical Statistics and Informatics, Faculty of Medicine, University of Belgrade, Belgrade, Serbia; ^3^ Clinic of Allergy and Immunology, University Clinical Center of Serbia, Belgrade, Serbia; ^4^ Faculty of Medicine, University of Belgrade, Belgrade, Serbia; ^5^ Institute of Rheumatology, Faculty of Medicine, University of Belgrade, Belgrade, Serbia; ^6^ Institute of Human Genetics, Faculty of Medicine, University of Belgrade, Belgrade, Serbia

**Keywords:** Epstein-Barr virus (EBV), systemic lupus erythematosus (SLE), marker, EA(D) IgG, anti-EBV antibodies, EBNA1, LMP1

## Abstract

**Introduction:**

The relationship between Systemic lupus erythematosus (SLE) and Epstein-Barr virus (EBV) infection has been suggested for decades, but the underlying mechanism of the EBV influence on SLE development remains to be elucidated.

**Methods:**

The goals of this research, which included 103 SLE patients and 99 controls, were to investigate the association of the parameters of EBV infection and SLE, to explore whether pooled demographic, clinical and EBV markers achieve a more significant effect on SLE development than each of them individually, and to evaluate EBV nuclear antigen 1 (EBNA1) and latent membrane protein 1 (LMP1) gene polymorphisms in isolates from SLE patients.

**Results:**

Comprehensive results related to serological, molecular and sequence markers of EBV infection in SLE patients demonstrated even 24 times higher possibility of having SLE if there is the presence of anti-EBV-EA(D) (early antigen) IgG antibodies (OR=24.086 95%CI OR=2.86-216.07, p=0.004). There was the same distribution of glucocorticoids (p=0.130), antimalarials (p=0.213), and immunosuppressives (p=0.712) in anti-EBV-EA(D) IgG positive and negative SLE patients. Further, higher anti-EBV-EA(D) IgG antibodies titers were identified as independent factors associated with lymphopenia, hematological SLE manifestation (OR=1.041, 95%CI OR=1.01-1.08, p=0.025, while a higher titer of anti-CA (viral capsid antigen) IgG antibodies (OR=1.015, 95%CI OR=1.01-1.03, p=0.019) and positive RF (rheumatoid factors) (OR=4.871, 95%CI OR=1.52-15.61, p=0.008) were identified as independent factors associated with alopecia within SLE. Finally, novel data on EBV EBNA1 and LMP1 gene polymorphisms in lupus are reported.

**Conclusion:**

The results support further investigation targeting EBV as a prognostic marker and therapeutic goal for lupus.

## 1 Introduction

Systemic lupus erythematosus (SLE) is a female predominant systemic autoimmune disease. It is characterized by the involvement of multiple organs and significant morbidity through pathogenic autoantibody production caused by the autoactivation of T and B cells. SLE includes repeated episodes of exacerbation, known as flares, and remission. Similar to other autoimmune disorders, the etiology of SLE still needs to be clarified entirely. The interplay between various genetic and environmental risk factors contributes to the onset and perpetuation of SLE. From external factors, infection, particularly Epstein-Barr virus (EBV) infection, has multiple potential roles in driving autoimmunity (1).

EBV is one of the most common human viruses. As a member of the Human Herpesviruses family, it latently infects up to 99% of the world’s population with the ability to survive in cells for life (2). With such unique capacities, it represents a constant challenge to the host. After the primary lytic infection of epithelial cells, EBV maintains latency in B lymphocytes with the expression of a limited number of genes defined with different types of latency (3). However, it occasionally reactivates the lytic cycle resulting in shifts between different sets of expressed genes (4). The humoral response includes the synthesis of antiviral antibodies against antigens of both the lytic and latent phases. Firstly, anti-EA (early antigen) and anti-CA (viral capsid antigen), during the first 3-4 weeks and at the time of the onset of the clinical symptoms, respectively (5). Although anti-CA IgG levels persist for life, the levels are higher during lytic infection (primary or reactivation) (6). On the other hand, EA IgG could be found in 85% of the acute infection, with usual persistence of 3 months, but with detectable levels years after primary infection in some cases (5). It is estimated that up to 30% of healthy subjects with a history of EBV have EA (D) IgG (5). Moreover, these antibodies could be detectable during reactivations or in immunocompromised patients (5, 6). Anti-EBNA-1 IgG is usually undetectable during the first month after the onset of clinical symptoms and is therefore indicative of past infection (5).

The relationship between SLE and EBV infection has been suggested for decades, but the underlying mechanism of the EBV influence on SLE progression remains to be elucidated. Several models have been proposed, among which antibody production through typical molecular mimicry and epitope spreading is the most important. The EBV genome encodes human homolog proteins such as latent protein, EBV nuclear antigen 1 (EBNA1), and the lytic protein, viral IL-10, that in SLE patients stimulate humoral and inflammatory immune responses (7). In particular, EBNA1 epitopes induce the production of autoantibodies against C1q and cross-react with SLE autoantigens, including Sm and Ro (7, 8). Based on the previously described knowledge, it was suggested that impaired control of EBV infection and cell-mediated immunity, with altered T cell function and the presence of multiple antibodies, leads to polyclonal expansion of EBV-infected B cells (9). Finally, autoreactive B cell activation during flares leads to frequent relapsing-remitting episodes of EBV infection, contributing to a vicious circle of repeated immune stimulations (10). Recent examples of more frequent EBV reactivations in SLE patients include the presence and elevation of serological markers CA IgG and EA IgG, higher frequencies of EBV-infected cells, higher viral loads and blood mononuclear cells, and increased expression of lytic genes (11). Moreover, in SLE patients, EBV-specific cytotoxic T and dendritic cells have reduced functionality, decreasing interferon levels after EBV infection (12, 13). The inverse relationship between decreased EBV lytic cycle responsive T cells and levels of anti-EBV antibodies is also followed by a negative correlation with the SLE disease activity index (SLEDAI) (14).

The balance that EBV establishes with the immune system and the host’s capacity to control cell proliferation in some EBV-associated diseases could be braked by the consequences influenced by specific variability of EBV genes (15, 16). In this regard, EBNA1 and latent membrane protein 1 (LMP1) genes are particularly interesting, with EBNA1 as the most prominently expressed EBV gene and LMP1 as the crucial viral oncogene. Their nucleotide variants and sequence specificities in previous studies showed potential contributions to the malignancy development (15, 17, 18). However, investigation of EBV gene variability and whether any mutation in viral genes contributes to SLE pathology has never been published before. Moreover, understanding this diversity in EBV-associated diseases revealed the possibility of predicting potential viral epitope targets to determine a treatment strategy (19).

Guided by the knowledge gaps and numerous unsolved hypotheses about SLE evolution, the goals of this research were set: a) to investigate the association between the respondents’ characteristics, including the parameters of EBV infection and SLE; b) to explore whether pooled markers achieve a more significant effect on SLE development than each of them individually, c) to evaluate EBV EBNA1 and LMP1 gene polymorphisms in isolates from SLE patients.

## 2 Materials and methods

### 2.1 Study design and participants

This prospective cohort study included 103 SLE patients diagnosed and treated at the Clinic for Allergology and Immunology University Clinical Center of Serbia and the Institute of Rheumatology, Belgrade, between June 2020 and November 2021. All SLE patients met at least 4 ACR SLE 1997 classification criteria (20). Patients with significant comorbidities (severe cardiac, pulmonary, and psychiatric diseases or dementia) and those unable to cooperate were excluded from the study. After detailed clinical interviews and physical examination of patients, all relevant demographic, clinical, and laboratory data were collected from their medical records. Disease activity was assessed using SLEDAI 2K scale (21) and Physician Global Assessment (PGA) (21), while fatigue was evaluated by the FACIT Fatigue Scale (version 4) (22). Patients with SLEDAI 2K≥6 were considered to have active SLE. Lupus Low Disease Activity State (LLDAS) was defined as SLEDAI-2K score ≤4, no activity in major organs, and PGA <1 (23, 24), while SLEDAI-2K score = 0 defined remission. The control group consisted of 99 participants. After a thorough interview and physical examination, we excluded those with positive personal or family histories of systemic autoimmune diseases and suggestive signs and symptoms of systemic autoimmune or active malignant disease. The exclusion criteria for both study cohorts were age under 18, unfeasibility to cooperate, and malignancies. All participants provided written informed consent. The study was performed in accordance with the Declaration of Helsinki and was approved by the Ethical Board of the Faculty of Medicine, University of Belgrade (No 1550/IX-14).

### 2.2 Samples

SLE patients’ serum and whole blood samples were collected at the Clinic for Allergology and Immunology University Clinical Center of Serbia and at the Institute of Rheumatology, Belgrade. Control group serum and whole blood samples were obtained from volunteers at the Institute of Rheumatology, Belgrade and the Institute of Microbiology and Immunology, Faculty of Medicine, University of Belgrade. After collecting 5 ml of blood in plain vacutainers, sera were separated by centrifugation. Plasma was also separated by centrifugation from 5 ml of ethylenediaminetetraacetic acid (EDTA) blood collected in vacutainer tubes. Two tubes (sera and plasma) from each patient were immediately tested or stored at -70°C until further analysis.

### 2.3 EBV serological testing

Anti-EBV antibodies against CA (IgG), CA (IgM), EA (IgG), EA (IgM), and EBNA1 (IgG) were identified and measured using commercial ELISAs according to manufacturer’s instructions in collected sera (Euroimmun, Lubeck, Germany). The assays used recombinant [EBNA1 and EA(D)] or purified (CA) antigens. Standard calibrators were used in each assay to calculate index values/optical density (OD) ratios, which serve as a quantitative measure of IgG antibody levels or a semi-quantitative measurement of IgM antibody levels. All assays met pre-determined quality control measures based on positive, negative, and blank controls. The positivity of IgG antibody presence was defined by a cut-off value of 20 relative units (RU/ml). For the sample values that were out of linearity defined by the used assays, re-measurement was done as following: samples were remeasured in a new test run at a dilution of e.g. 1:400 and results in RU/ml read from the calibration curve were multiplied by a factor 4. The positivity of IgM antibody presence was defined as OD ratio ≥1.1. Absorbances were recorded on a Multiscan FC microplate reader (Thermo Scientific, Massachusetts, USA) using a wavelength of 405 nm with background subtraction at 650 nm.

### 2.4 EBV DNA detection

Viral DNA was isolated from 200 μL plasma using a PureLink Genomic DNA Mini Kit (Invitrogen by Thermo Fisher Scientific, Massachusetts, USA) according to the manufacturer’s instructions. Two hundred and two DNA isolates were further used in a nested-PCR method to amplify two EBV genes: EBNA1 and LMP1. Amplifications of the C terminus of EBNA1 and C terminus of LMP1 were performed by nested PCRs as described previously (25), using primers that were reported by Lorenzetti et al. and Li et al. (26, 27).

### 2.5 EBV EBNA1 and LMP1 carboxy-terminal regions sequencing with sequence analysis

For purification, performing cycle sequencing reactions and sequencing of EBNA1 and LMP1, both sense and antisense strands, principles described before were used (25).

LMP1 and EBNA1 nucleotide sequences 506-bp and 329-bp long were separately aligned and compared with a reference sequence in Bioedit 7.0.5.3 software (28). EBNA1 subtypes and sub-variants were defined after inspection of signature amino acid changes at the following positions: 471, 475, 476, 479, 487, 492, 499, 500, 502, 517, 520, 524, 525, 528, and 533. Characterization of LMP1 variants was performed using the same software by examination of amino acid changes described by Edwards et al. (29). The sequence used in both alignments as the reference was downloaded from the GenBank/EMBL7DDBJ database under the accession number V01555.

### 2.6 Data analysis

Numerical data were described with the arithmetic mean and standard deviation or median and interquartile range (IQR), depending on the data distribution. Normal distribution was evaluated by mathematical (Kolmogorov-Smirnov and Shapiro-Wilk test, skewness and kurtosis, and coefficient of variation) and graphical methods (histogram, box-plot, Q-Q diagram). Categorical variables were presented as absolute and relative numbers in n (%). Student t-test for two independent samples or Mann-Whitney U test was used to compare numerical data between study cohorts, depending on the data distribution. The chi-square test was used to test the difference in the distribution of categories of two independent samples.

To evaluate all possible factors influencing a person’s probability of having SLE, univariate logistic regression analysis was performed first, with multivariate analysis after that with significant variables from the previous analysis in the model. Variance Inflation Factor (VIF) multicollinearity testing method and correlation coefficients were used. All variables with VIF>5 were eliminated from the multivariate model. Also, according to the co-variance matrix and correlation coefficients, one of two correlated variables with a lower p-value in univariate logistic regression was eliminated. Forward Wald regression method was applied, and only the last step was presented in the results. The odds ratio (OR), 95% confidence interval of odds ratio (95% CI OR), and p-value were reported. Complete statistical analysis was performed in SPSS Statistics for Windows Version 26.0 (IBM Corp. Armonk, NY).

The sample size was calculated by G Power 3.1.9.2 for the major outcome – the presence of anti-EBV-EA(D) IgG antibodies in SLE patients and control according to previously known data (7) and for the effect size of 0.3, error of the I type (α) of 0.05, and statistical power of 0.8. It was obtained that 88 observation units per study group would be enough to determine the differences between them.

## 3 Results

### 3.1 Characteristics of the study cohorts

This study included 103 SLE patients, mostly women (91.3%), with an average age of 45.42 ± 12.9 years. The most common manifestations were lymphopenia (50%), then alopecia (39%), rash (32%), arthritis (32%), and leucopenia (32%). Lupus nephritis was present in 15%, neutropenia in 13%, mucosal ulcerations in 12%, and thrombocytopenia in 11%. Almost 40% of SLE patients had secondary Sjogren syndrome, and less than 10% had secondary antiphospholipid syndrome. Of 99 participants in the control group, there were 88% women with an average age of 55.43 ± 13.62 years. More detailed demographic, clinical and laboratory characteristics of study cohorts are shown in **Table 1**. SLE patients were treated with corticosteroids (87%), antimalarials (83%), immunosuppressants (29%), and pulse therapy (13%).

**Table 1:** Baseline characteristics of the study cohorts.

Characteristic	Group		p^*^
	SLE n=103	Controls n=99	
Age (years), mean ± sd	45.43 ± 12.90	55.43 ± 13.62	**<0.001£**
Gender, n (%)			
Male	9 (8.7)	12 (12.1)	0.431§
Female	94 (91.3)	87 (87.9)
Duration of SLE (years), med (IQR)	6.0 (3.0-11.0)	/	NA
BMI, mean ± sd	25.93 ± 5.25	27.18 ± 4.54	0.074£
Smoking status, n (%)			
Smoker	60 (60.0)	39 (39.4)	**0.004§**
Non-smoker	40 (40.0)	60 (60.6)
Smoking duration (years), med (IQR)	16.0 (10.0-30.0)	25.0 (15.0-30.0)	**<0.001¥**
Hashimoto thyroiditis, n (%)	17 (16.7)	15 (15.2%)	0.769§
Secondary Sjogren`s syndrome, n (%)	39 (37.9)	0 (0.0)	**<0.001§**
Antiphospholipid syndrome, n (%)	9 (8.7)	0 (0.0)	**0.003§**
Comorbidities, n (%)			
HTA	44 (42.7)	47 (47.5%)	0.497§
DM	7 (6.8)	11 (11.1%)	0.282§
Cardiovascular events (CVI, TIA, AIM, AP), n (%)	9 (8.7)	17 (17.2%)	0.074§
No of ACR 1997 criteria, med (IQR)	6.0 (5.0-6.0)	/	NA
Immunological parameters			
ANA, med (IQR)	640.0 (40.0-640.0)	/	NA
Anti-dsDNA positivity, n (%)	19 (37.3)	/	NA
Anti-SSA positivity, n (%)	27 (54.0)	/	NA
Anti-Sm positivity, n (%)	19 (37.3)	/	NA
aCL-IgM positivity, n (%)	7 (14.0)	/	NA
aCL-IgG positivity, n (%)	8 (16.0)	/	NA
Anti- β2-GPI IgG, n (%)	7 (7.4)	/	NA
Anti-β2-GPI IgM, n (%)	7 (7.4)	/	NA
RF positivity, n (%)	14 (28.6)	/	NA
C3, mean ± sd	0.81 ± 0.27	/	NA
C4, med (IQR)	0.12 (0.07-0.21)	/	NA
Total IgG, mean ± sd	13.91 ± 5.20	/	NA
Currently active SLE manifestations, n (%)			
Rash	33 (32.0)	/	NA
Arthritis	33 (32.0)	/	NA
Mucosal ulcerations	12 (11.7)	/	NA
Alopecia	40 (38.8)	/	NA
Serositis (pleuritis)	2 (1.9)	/	NA
Lupus nephritis	15 (14.6)	/	NA
NPSLE	4 (3.9)	/	NA
Leucopenia	33 (32.0)	/	NA
Neutropenia	13 (13.1)	/	NA
Lymphopenia	50 (50.0)	/	NA
Thrombocytopenia	11 (10.8)	/	NA
FACIT score	35.51 ± 12.05	/	NA
SLICC/ACR Damage Index	0.0 (0.0-6.0)	/	NA
Current SLE therapy			
Corticosteroids, n (%)	90 (87.4)	/	NA
Prednisone daily dose (mg), med (IQR)	12.5 (6.87-20.0)	/	NA
Hydroxychloroquine, n (%)	86 (83.5)	/	NA
Immunosuppressives (AZA, MTX, MMF)	30 (29.1)	/	NA
Pulse therapy (Cyclophosphamide and/or Glucocorticoids)	12 (12.7)	/	NA
Laboratory assessment, med (IQR)			
ESR	22.0 (12.2-38.0)	18.0 (12.0-24.5)	0.066¥
CRP	2.8 (1.23-7.6)	2.2 (1.2-5.1)	0.169¥
NLR	2.1 (1.4-3.4)	2.2 (1.7-2.9)	0.497¥
Disease activity assessment, med (IQR)			
SLEDAI 2K	5.0 (2.0-10.0)	/	NA
Clinical SLEDAI 2K	3.0 (0.0-8.0)	/	NA
PGA, med (min-max)	0.84 (0.15-1.38)	/	NA
Disease activity, n (%)			
Active SLE	41 (39.8)	/	NA
LLDAS/remission	62 (60.2)	/

*for the level of significance of 0.05, according to Student t-test £, Chi-square test §, Mann-Whitney U test ¥, NA, not applicable.

If the p value is in bold, it means that statistical significance is achieved.

SLE, systemic lupus erythematosus; BMI, body mass index; APS, antiphospholipid syndrome; HTA, hypertension; DM, diabetes mellitus; CVI, cerebrovascular insult; TIA, transitory ischemic attack; AMI, acute myocardial infarction; AP, angina pectoris; NPSLE, neuropsychiatric SLE; RF, rheumatoid factor; AZA, Azathioprine; MTX, Methotrexate; MMF, Mycophenolate mofetil; ESR, estimated sedimentation rate; CRP, C-reactive protein; NLR, neutrophil-to-lymphocyte ratio.

### 3.2 EBV infection status in SLE patients and controls

The prevalence of EBV DNA (EBNA1 and/or LMP1 gene) was 11% in the plasma of SLE patients, without statistically significant difference in comparison with controls (p=0.893). Anti-EBV-CA IgM, anti-EBV-EA(D) IgG, and anti-EBV-EA(D) IgM antibodies were more prevalent in SLE patients than in controls (p<0.001, p<0.001, and p=0.008, respectively). The status of EBV infection was determined based on the results from both EBV DNA and anti-EBV antibody testing. The active EBV infection was declared if EBV DNA and/or anti-EBV IgM antibodies were present. SLE patients more often had active EBV infection than controls (42% vs 18%, p<0.001) (**Table 2**). SLE patients with latent EBV infection were significantly more often treated with immunosuppressive drugs than those with active infection (37% vs. 19%, p=0.047), while the distribution of other medications (corticosteroids, antimalarials, pulse therapy) was the same between SLE patients with active and latent EBV infection (p=0.797, p=0.306, and p=0.773, respectively) (Available in **Supplementary Material Table 1**).

**Table 2:** Prevalence of EBV DNA, anti-EBV antibodies and active EBV infection.

EBV DNA and anti-EBV antibodies, n (%)	Group		p^*^
	SLE n=103	Control n=99	
EBV DNA	11 (10.7)	10 (10.1)	0.893§
Anti-EBV-EBNA1 IgG	99 (96.1)	97 (98.0)	0.436§
Anti-EBV-CA IgG	103 (100.0)	98 (99.0)	0.490€
Anti-EBV-CA IgM	26 (25.2)	3 (3.0)	**<0.001§**
Anti-EBV-EA(D) IgG	35 (34.0)	5 (5.1)	**<0.001§**
Anti-EBV-EA(D) IgM	22 (21.4)	8 (8.1)	**0.008§**
EBV infection status, n (%)			
active	43 (41.7)	18 (18.2)	**<0.001**
latent	60 (58.3)	81 (81.8)

*for the level of significance of 0.05, according to Chi-square test§ or Fisher’s exact test€.

If the p value is in bold, it means that statistical significance is achieved.

**Figure 1:**
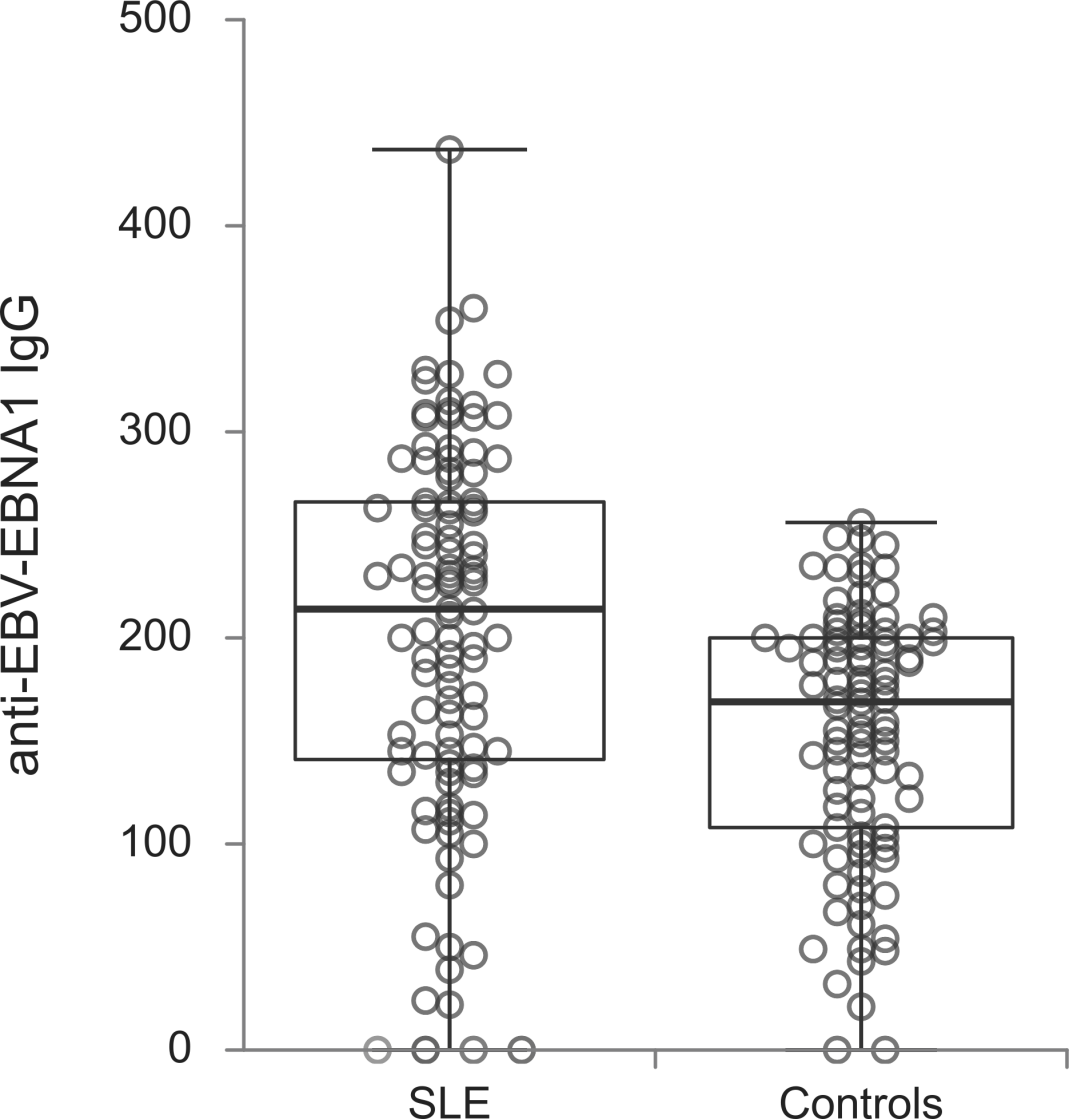
Anti-EBV-EBNA1 IgG antibodies in SLE patients and controls (p<0.001).

**Figure 2:**
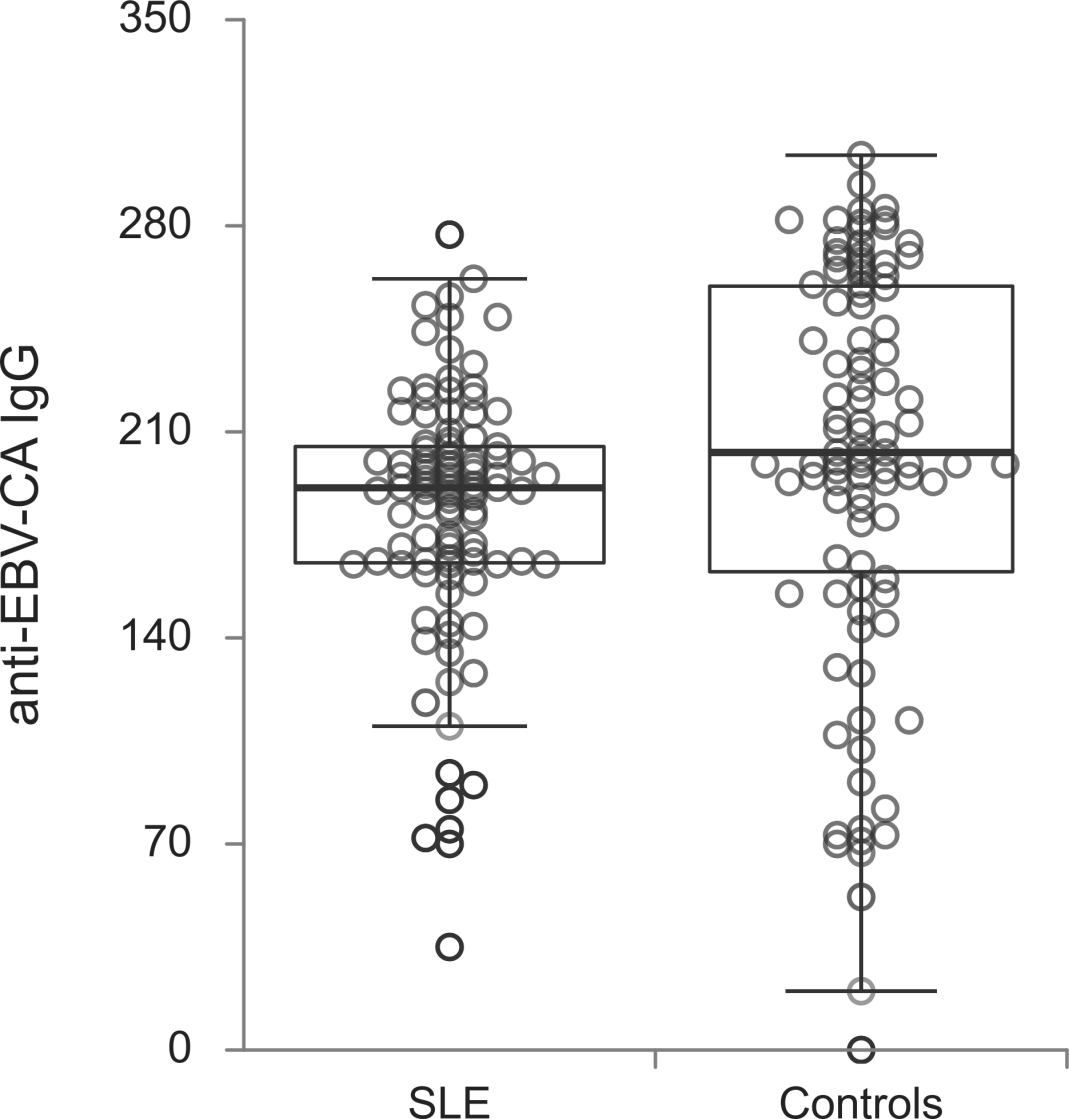
Anti-EBV-CA IgG antibodies in SLE patients and controls (p=0.003).

**Figure 3:**
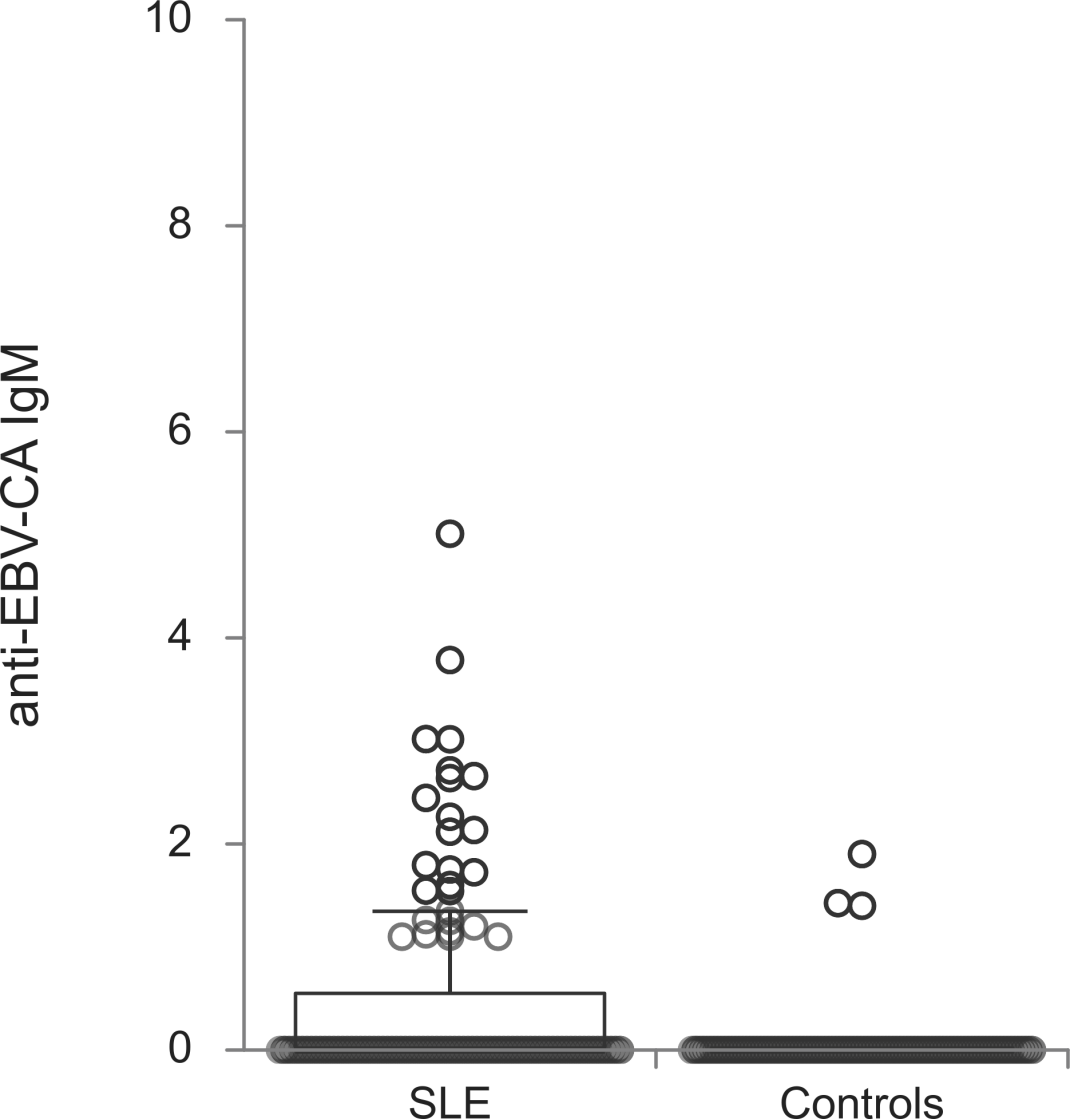
Anti-EBV-CA IgM antibodies in SLE patients and controls (p<0.001).

**Figure 4:**
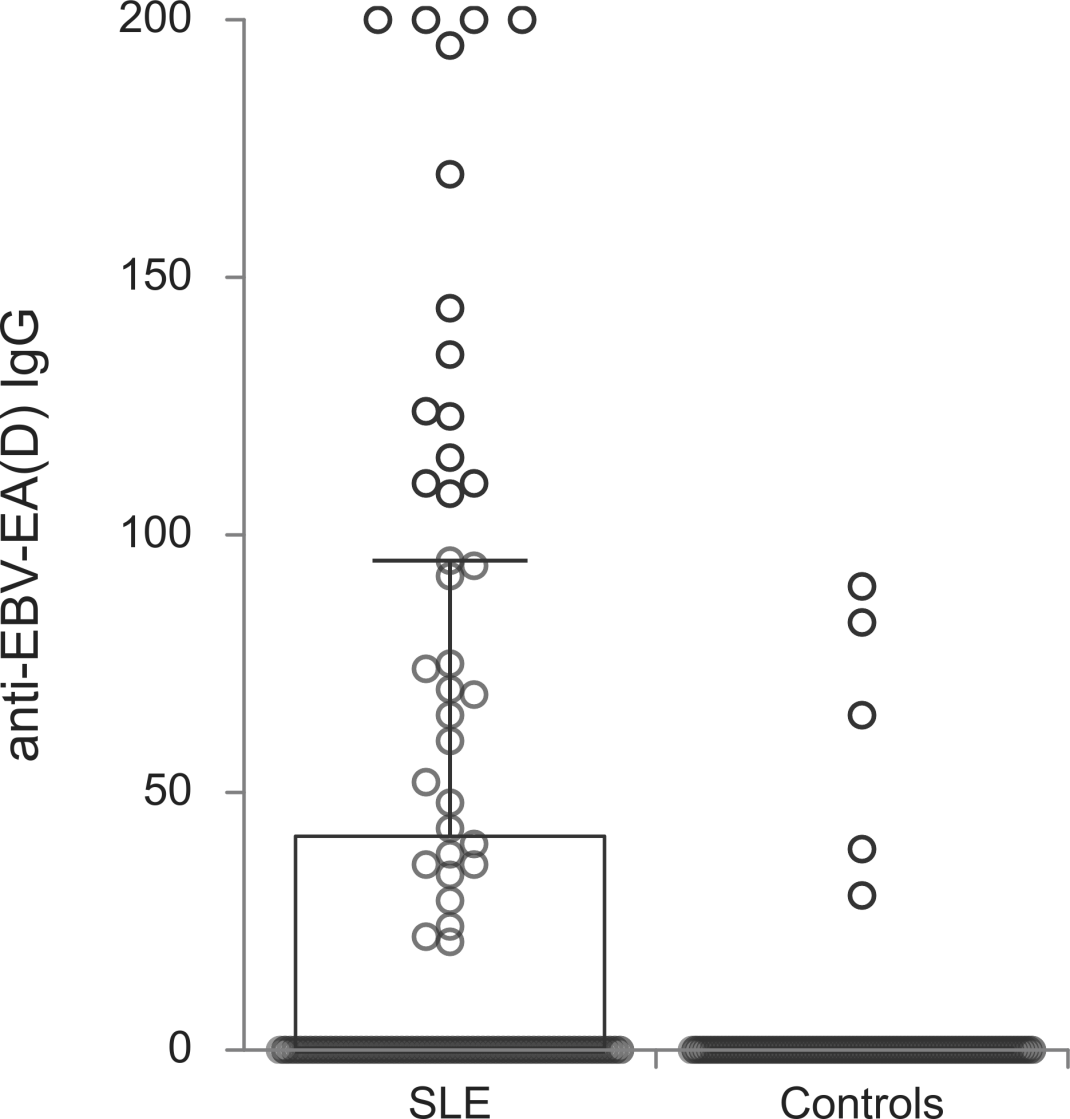
Anti-EBV-EA(D) IgG antibodies in SLE patients and controls (p<0.001).

**Figure 5:**
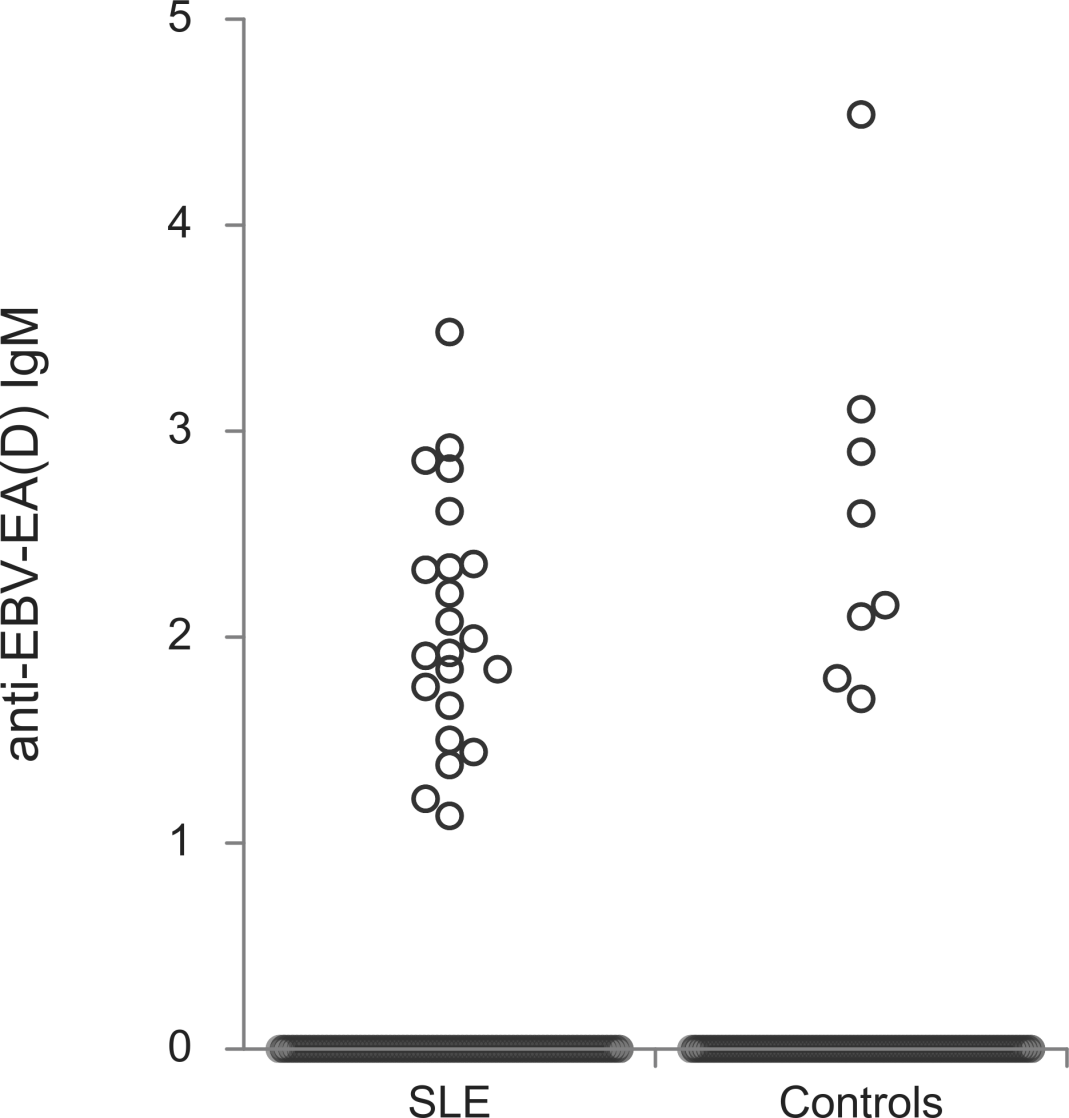
Anti-EBV-EA(D) IgM antibodies in SLE patients and controls (p=0.012).

The titers of all evaluated anti-EBV antibodies in SLE patients and controls were further compared (**Figures 1**–**5**). SLE patients had significantly higher titers of anti-EBV-EBNA1 IgG, anti-EBV-CA IgM, anti-EBV-EA(D) IgG, anti-EBV-EA(D) IgM antibodies than controls (p<0.001, p<0.001, p<0.001, and p=0.012, respectively), while the titer of anti-EBV-CA IgG antibodies was significantly higher in controls than in SLE patients (p=0.003).

### 3.3 Evaluation of factors associated with SLE

All applicable EBV and clinicodemographic parameters were evaluated for defined outcomes (presence of SLE, the active form of SLE disease, and different clinical manifestations of SLE: rash, arthritis, ulcerations, alopecia, nephritis, leucopenia, lymphopenia, thrombocytopenia). Univariate, then multivariate regression analysis was performed. All variables with p<0.05 in univariate regression were included in the multivariate model taking into consideration collinearity. The complete analysis is available in the **Supplementary Material Tables 2**-**12**.

#### 3.3.1 Baseline characteristics and markers of EBV infection in association with systemic lupus erythematosus

According to multivariate regression modeling, younger age (OR=0.814, 95%CI OR=0.74-0.89, p<0.001), the presence of anti-EBV-EA(D) IgG antibodies (OR=24.086, 95%CI OR=2.68-216.07, p=0.004), higher titers of anti-EBNA1 IgG antibodies (OR=1.023, 95%CI OR=1.01-1.03 p<0.001) and anti-EBV-CA IgM antibodies (OR=3.105, 95%CI OR=1.07-8.98, p=0.036), and lower titer of anti-EBV-CA IgG antibodies (OR=0.984, 95%CI OR=0.97-0.99, p=0.013) were independently associated with the presence of systemic lupus erythematosus (**Table 3**).

**Table 3:** Factors associated with Systemic lupus erythematosus and its manifestations.

Factors associated with Systemic lupus erythematosus (step 5)			
Factor	OR	95%CI OR	p^*^
Age	0.814	0.74-0.89	**<0.001**
Presence of anti-EBV-EA(D) IgG	24.086	2.68-216.07	**0.004**
Anti-EBV-EBNA1 IgG Ab titer	1.023	1.01-1.03	**<0.001**
Anti-EBV-CA IgM Ab titer	3.105	1.07-8.98	**0.036**
Anti-EBV-CA IgG IgG titer	0.984	0.97-0.99	**0.013**
Factors associated with active form of Systemic lupus erythematosus (step 3)			
Number of positive ACR criteria	1.819	1.08-3.07	**0.025**
ESR	1.037	1.01-1.07	**0.020**
C3	0.036	0.01-0.46	**0.011**
Rash (step 2)			
Positive RF	3.500	1.15-10.66	**0.028**
C3	0.056	0.01-0.38	**0.003**
Arthritis (step 2)			
Number of positive ACR criteria	1.599	1.08-2.36	**0.018**
ESR	1.033	1.01-1.06	**0.005**
Mucosal ulcerations (step 2)			
Secondary Sjogren syndrome	5.524	1.16-26.30	**0.032**
Number of positive ACR criteria	2.162	1.40-3.35	**0.001**
Alopecia (step 2)			
Anti-EBV-CA IgG Ab titer	1.015	1.01-1.03	**0.019**
Positive RF	4.871	1.52-15.61	**0.008**
Lupus nephritis (step 2)			
Presence of anti- β2 IgG Ab	6.649	1.21-36.40	**0.029**
Anti-dsDNA Ab titer	1.004	1.00-1.01	**0.001**
Lymphopenia (step 3)			
Smoking duration	0.835	0.73-0.96	**0.009**
NLR	6.443	2.06-20.16	**0.001**
antiEBV-EA(D) IgG Ab titer	1.043	1.01-1.08	**0.025**
Thrombocytopenia (step 1)			
Smoking duration	0.854	0.74-0.99	**0.036**

*for the level of significance of 0.05, according to Forward: Wald logistic regression method.

If the p value is in bold, it means that statistical significance is achieved.

#### 3.3.2 Association between baseline characteristics and markers of EBV infection and active SLE disease

According to multivariate regression modeling, a greater number of positive criteria (OR=1.819, 95%CI OR=1.08-3.07, p=0.025), higher ESR (OR=1.037, 05%CI OR=1.01-1.07, p=0.020), and lower C3 complement level (OR=0.036, 95% CI OR=0.01-0.46, p=0.011) were independent factors associated with active SLE disease (**Table 3**).

#### 3.3.3 Association between baseline characteristics and markers of EBV infection and lupus manifestations

##### 3.3.3.1 Rash

Lower C3 level (OR=0.056, 95%CI OR=0.01-0.38, p=0.003) and positive rheumatoid factor (OR=3.500, 95%CI OR=1.15-10.66, p=0.028) were independent factors associated with rash lupus manifestation (**Table 3**).

##### 3.3.3.2 Arthritis

Greater number of positive criteria (OR=1.599, 95%CI OR=1.08-2.36, p=0.018) and higher ESR (OR=1.033, 95%CI OR=1.01-1.06, p=0.005) were independent factors associated with arthritis within SLE (**Table 3**).

##### 3.3.3.3 Mucosal ulcerations

Greater number of positive ACR criteria (OR=2.162, 95%CI OR=1.40-3.35, p=0.001) and presence of secondary Sjogren syndrome (OR=5.524, 95%CI OR=1.16-26.30, p=0.032) were factors independently associated with mucosal ulcerations within SLE (**Table 3**).

##### 3.3.3.4 Alopecia

Higher titer of anti-EBV-CA IgG antibodies (OR=1.015, 95%CI OR=1.01-1.03, p=0.019) and positive RF (OR=4.871, 95%CI OR=1.52-15.61, p=0.008) were independent factors related to alopecia within SLE (**Table 3**).

##### 3.3.3.5 Lupus nephritis

Positive anti-b2-GP I IgG (OR=6.649, 95%CI OR=1.21-36.40, p=0.029) and higher titer of anti-dsDNA (OR=1.004, 95%CI OR=1.00-1.01, p=0.001) were independent factors associated with lupus nephritis (**Table 3**).

##### 3.3.3.6 Leucopenia

The presence of anti-SSA antibodies was the only factor potentially associated with leucopenia (OR=4.676, 95%CI OR=1.18-18.51, p=0.028).

##### 3.3.3.7 Lymphopenia

Shorter smoking duration (OR=0.835, 95%CI OR=0.73-0.96, p=0.009), higher neutrophil-to-lymphocyte ratio (NLR) (OR=6.443, 95%CI OR=2.06-20.16, p=0.001), and higher titer of anti-EBV-EA(D) IgG antibodies (OR=1.041, 95%CI R=1.01-1.08, p=0.025) were independent factors associated with Lymphopenia (**Table 3**).

##### 3.3.3.8 Thrombocytopenia

Shorter smoking duration (OR=0.854, 95%CI OR=0.74-0.99, p=0.036) was an independent factor associated with thrombocytopenia (**Table 3**).

### 3.4 Variations of EBV EBNA1 and LMP1 carboxy-terminal regions sequences

Eight sequences of the EBNA1 gene fragment (coordinates 109261-109590) from 8 SLE patients were analyzed and compared with the B95-8 prototype strain. Inspection included signature amino acid changes at the following positions: 471, 475, 476, 479, 487, 492, 499, 500, 502, 517, 520, 524, 525, 528, and 533. Based on amino acid substitutions, isolates were grouped into two prototype subtypes, P-Ala and P-Thr (**Table 4**). Since none of the obtained sequences fully corresponded to the prototype sequences, further analysis of additional nucleotide variability and amino acid substitutions was performed. Three subvariants (sv) were identified: P-Ala-sv-2 and -3; P-Thr-sv-7. All three subvariants were previously found in Serbian isolates, however, P-Ala-sv-3 and P-Thr-sv-7 were described as newly identified (18). P-Ala-sv-3 differed from P-Ala-sv-2 in position 499, where the new subvariant retained the same nucleotide combination as the prototype sequence. P-Thr-sv-7 was characterized by four changes compared to the prototype sequence (positions 476, 487, 492, and 524) or two changes compared to the P-Thr sequence (499 and 520).

**Table 4:** EBNA1 C-terminal nucleotide and amino acid changes found in EBV isolates in SLE patients.

	B95-8^a^ (P-Ala)	P-Ala-sv-2	P-Ala-sv-3^b^	P-thr-sv-7^b^
Locus				
**471**	CAA Gln			
**476**	CCG Pro			CAG Gln
**483**	GAA Glu			
**487**	GCT Ala			ACT Thr
**492**	AGT Ser			TGT Cys
**499**	GAC Asp	GAA Glu		
**502**	ACT Thr			
**520**	CTA Leu			
**524**	ACT Thr	GTT Val	GTT Val	ATT Ile
**529**	CCA Pro			
**533**	CTT Leu			
**Number of isolates** (every isolate originated from one SLE patient)	–	3	2	3
**Clinical presentation of SLE found in at least one patient**		Rash, Arthritis, Lymphopenia, Leucopenia	Arthritis, lymphopenia, Thrombocytopenia	Rash, Arthritis, lymphopenia, Leucopenia, Thrombocytopenia
**Total** number of isolates (patients)	8			

^a^ Prototype sequence (represents the P-Ala-subtype).

^b^ New subvariants: P-Ala-sv-3, P-Thr-sv-7.

Eight sequences of the LMP1 gene fragment (coordinates 168719-168213) from 8 SLE patients were aligned and compared with the B95-8 prototype strain. Characterization of LMP1 variants was performed using by well-known examination of amino acid changes described by Edwards et al. (29). Due to divergence from the reference sequence, presence of deletions, number of 11-amino acid repeats and total amino acid changes, isolates were divided into four variants: B95-8, North Carolina, Mediterranean and China1 (**Table 5**).

**Table 5:** Distribution of three LMP1 characteristics in EBV isolates in SLE patients.

		LMP1 variant	Number of LMP1 33-bp tandem repeat units^b^	LMP1 deletion^c^
Number of isolates (every isolate originated from one SLE patient)	2	B95-8^a^	4.5	–
	3	North Carolina (NC)	4 (2 isolates)	–
			3 (1 isolate)	–
	1	Medditerranean (Med)	4	–
	2	China1	3 (1 isolate)	30-bp
			7 (1 isolate)	
Total number of isolates (patients)	8			

^a^Prototype sequence.

^b^11-amino acid/33-bp repeats located between amino acids 250 and 308. The prototype B95-8 sequence has four perfect repeats with a disruption of 5 amino acids between the second and the third repeat.

^c^Specific 10-amino acid/30-bp deletion (spanning codons 346-355).

## 4 Discussion

After numerous attempts to prove a cause-and-effect relationship between EBV infection and SLE, more data on EBV-dependent features still need to be included for this evidence (30). The only fact is that the association between SLE and EBV increased reactivation due to insufficient latency regulation or enhanced transition from latent to lytic phase (30, 31). This confirmation was established by a few dozen studies based on detecting EBV infection markers in lupus patients (9). However, most studies measured a limited number of viral markers, quite often without a control group to compare findings. Thus, the latest publications did not include IgM antibodies to CA and EA, free viral DNA or gene sequence variability (7). Moreover, European research was limited primarily to Denmark and Italy, with rare exceptions from France, Netherlands and Hungary (9). To our knowledge, comprehensive analysis of EBV markers in lupus patients and their potential relationship with characteristics, different clinical presentations of SLE or therapeutic modalities is rare among literature data. Therefore, identifying independent EBV factors for SLE and our study’s first molecular analysis of EBV genes represent a valuable contribution to this still under-investigated scientific field.

It is known that occasional EBV reactivations can be diagnosed serologically and by molecular detection of viral DNA. Although the specific serological interpretation of EBV reactivation in SLE patients may be missed because of a possible reflection of a non-specific antibody increase due to an autoimmune background, the available results say otherwise. The antibody responses towards other herpesviruses, including cytomegalovirus (CMV) and herpes simplex virus (HSV), are similar between SLE patients and controls (30). Interestingly, however, the dominant EBV latency type maintained during lupus could not be clearly defined using commonly known criteria. In particular, the combination of BZLF1 and LMP1 in SLE patients probably represents dysregulated latent gene expression in the direction of intermediate latency form between latency II and III (30). On the other hand, EBV reactivation in these patients does not have to be a consequence of immune dysregulation but perhaps immunosuppression (11).

This research emphasized the hypothesis that the immune response to EBV activity represents an important element in the sequence of events that follows the clinical course of SLE. According to the results, there is undoubtedly a highly significant EBV activity compared to the control group. Firstly, anti-EBV-CA IgM, anti-EBV-EA(D) IgG, and anti-EBV-EA(D) IgM antibodies were more prevalent in SLE patients than controls. Moreover, all of the mentioned antibodies together with anti-EBV-EBNA1 IgG had also significantly higher titers in SLE patients than controls. Previous research showed similar results, including prospective investigation of SLE disease transition (7, 11). Based on all processed viral parameters, active EBV infection was more often in the lupus group than in the control group, corroborating previous descriptions of the delicate relationship between the virus and SLE pathogenesis (7, 10, 11). Besides SLE, our research group obtained similar results in patients with rheumatoid arthritis (RA), another systemic autoimmune disease. Active or recent EBV infection was proven in the same percentage among RA patients (42%) and more prevalent than controls (32). Finally, approximately equal seroprevalence of anti-EBV-EBNA1-IgG and anti-EBV-CA IgG antibodies in both study groups showed the same level of prior EBV exposure. These findings suggested the specificity of selected serological markers that proved viral activity in SLE patients.

EBV, in particular EBNA1, is a candidate for the heteroimmune response (11). According to the hypothesis, this response represents the substrate for generating SLE autoantibodies. Thus anti-EBV-EBNA1 IgG antibodies should be present at a higher rate in lupus patients (31). However, very few of the latest studies dealing only with seroprevalence, have managed to show this (9, 31, 33). Their authors suggested that the anti-EBNA1 response independently carries a risk for SLE which was documented with the higher prevalence of anti-EBNA1 antibodies in lupus patients. On the other hand, more publications including one of the latest meta-analyses nor reach this conclusion (9). Following the recent findings from Laurynenka et al. (9, 31, 33) and their suggestion that anti-EBNA1 heteroimmune response is a foundation from which pathogenic SLE autoimmunity develops, our results could be an important contributor to this theory. Although in the subjects from our study, regardless of the presence of SLE, anti-EBNA1 IgG seroprevalence was equal, their titers were significantly higher in lupus patients. Thus, these additional data on higher titer values could be a consequence of progressed anti-EBNA1 response, its cross-reaction with SLE autoantigens led by molecular mimicry, autoantibody epitope spreading and development of clinically manifested lupus (9, 31, 33).

The reports about the seroprevalence of anti-EBV-CA IgG between lupus patients and controls are conflicting. Our study showed the same level of prior EBV exposure between groups, which was in concordance with publications from Filipinos, India, the USA and more (7, 34, 35). On the other hand, some literature data described the higher prevalence of anti-EBV-CA IgG and its association with the development of SLE (9, 11). The authors even noted the potential influence of race on this association (36). Regardless of the mentioned differences in seroprevalence, most works including our, were uniform about the titer results with proved significant differences in the anti-EBV-CA IgG titers in SLE compared to the control groups (7, 11). It is known that EBV reactivation increases anti-EBV-CA IgG levels suggesting a more frequent and significant uncontrolled viral replication in lupus compared to the control group (6). However in other systemic autoimmune diseases the case was not the same, and our research group did not prove the mentioned difference in the anti-EBV-CA IgG titers when RA patients were taken into consideration and compared with a control group (32).

One of the most noticeable results confirmed the strong association between anti-EBV-EA(D) IgG antibodies and SLE. Thus, this study, for the first time, revealed a 24 times higher possibility of having SLE, if there is a presence of anti-EBV-EA(D) IgG antibodies. According to our knowledge, only one study so far obtained a higher index of association between EBV infection and autoimmune disease based on serological EBV status. Unlike ours, it was a huge longitudinal study that proved a 32-fold increase in multiple sclerosis risk after infection with EBV (37). In addition, younger age, higher titers of anti-EBNA1 IgG antibodies and lower titers of anti-EBV-CA IgG antibodies in our study were also found to be independently associated with the presence of lupus, but with negligible OR values when compared with anti-EBV-EA(D) IgG antibodies. On the basis of this result, it could become clear that the presence of anti-EBV-EA(D) IgG antibodies is a property that characterizes lupus. This probably reflected the chronic production of antibodies against cells expressing EA(D) and released EA(D) from lysed EBV-infected cells (38). Due to different types of infected cells, enhanced production of anti-EA(D) antibodies includes not only IgG (lymphocytes) but also IgA (epithelial cells), in addition to autoantibodies as EA(D) bound to dsDNA functions as EBV-DNA polymerase (38). More anti-EA(D) antibody types could suggest disseminated infection with a higher capacity to induce cell lysis and consequential autoimmunity. In addition to this, it is important to mention an apparent contradiction in how the treatment of SLE, which is based on immunosuppression, could be successful against a virus-induced autoimmune disease. This finding was also obtained in our study. The presence and elevation of anti-EA(D) IgG antibodies were independent of the applied type of immunosuppressive therapy. Supported by previous publications also, this paradox might be explained by the biology of EBV, its tropism for B lymphocytes and the capacity of immunosuppression to limit the spreading of the virus and infected B cells in addition to its alleviation of inflammation (38–42). Relying on the suggestions from the literature data of the importance of anti-EBV-EA(D) IgG for discrimination of SLE patients, together with results from our study, it is indicative that anti-EBV-EA(D) IgG could be considered as a marker of the presence of the disease (43, 44). Moreover, it could also be used to design future targeted therapy, as mentioned in the literature data (7, 45). One of the most recent research about SLE therapy identified blocking EBV lytic phase switching as a critical (8).

Interestingly, some researchers have even demonstrated an association between EBV activity and the severity of lupus disease (7, 46–48). Others failed to find any association between EBV antibody profile, viral load, and clinical manifestations (12, 35). By analyzing the association between different EBV infection markers and individual clinical presentations of lupus patients from Serbia, this study identified viral serological predictors for alopecia and specific hematological SLE manifestation for the first time. In particular, only one RU/ml higher titer of anti-EBV-EA(D) IgG antibodies carries a 4% higher chance for lymphopenia. Although the shorter smoking duration and higher NLR were also factors associated with lymphopenia, elevated anti-EBV-EA(D) IgG titers could be helpful during the laboratory monitoring of lupus and indicate the possibility of developing a specific clinical manifestation. As this result was not reported earlier, properly explaining the underlying mechanism could be difficult. However, it might be essential to interpret it together with the results of Wood et al. (7). In one of their latest paper, anti-EBV-EA IgG antibodies were higher when expression of interferon (IFN) and inflammatory or lymphoid and monocyte responses were higher. In addition, those patients with higher anti-EBV-EA(D) IgG responses were more likely to have renal involvement. Another publication that described this potential association with lupus nephritis detected the expression of EBV genes in renal tissue (49). Finally, in our study, a higher titer of anti-CA IgG antibodies and positive RF revealed independent factors related to alopecia within SLE. This finding should be analyzed in further investigations also.

The results, including the interplay between smoking and EBV in autoimmunity development, are scarce and conflicting. In those reported in multiple sclerosis (MS) patients, there are indications of competing antagonism, where mentioned exposures compete to affect the outcome (50). This study did not reveal any similar findings. However, shorter smoking duration was shown as an independent factor associated with lymphopenia in lupus patients.

According to the last systemic review with meta-analysis, only seven studies tested and compared EBV DNA presence between SLE and the control group (9). In addition to high heterogeneity, the positive rate for EBV DNA in SLE and control group was 55.1% and 20.7%, respectively. This difference was not described as significant due to the small number of studies, participants and heterogeneity. Results from our study did not reveal any difference between patients and healthy participants, as it was also published lately by Han et al. (46). Many authors proved a higher viral load in lupus of up to 40-fold greater increase regardless of immunosuppressive therapy (38). On the other hand, some authors did not find any viral genome at all (51). Thus, the large discrepancy in the stratification of SLE patients and the selection of samples for DNA testing must be addressed. Unfortunately, previous reports refer to peripheral B cells, PBMC and even sera (7, 38, 46, 48). Unlike them, our study tested viral DNA from free cell blood compartments depicting the amount of virus released during active replication and tendentially eliminating the amount that rests in latency. It was estimated that healthy carriers have approximately 1 to 50 infected cells per million leukocytes or 1 to 30 copies of EBV DNA per million leukocytes in whole blood. Thus, besides B cells or PBMC samples, any biopsy tissue may contain B lymphocytes and harbor amplifiable EBV DNA, too (52).

In our small group of positive EBV DNA isolates from SLE patients, we reported characteristics of EBNA1 and LMP1 gene sequences as an additional novelty. In need of more data on EBV gene sequences in lupus, reports on EBV gene sequences in other systemic autoimmune diseases are also extremely rare. They could be only found in RA or multiple sclerosis (MS) (19, 53, 54). In addition, those reports included only EBNA1 and EBNA2 variants. Similar to our study, previous authors did not prove the correlation of EBV variability with the risk of autoimmune disease (19, 54). However, some assumptions exist that specific virus genetic variants contribute to autoimmune development (53). EBNA1 was described as an unusual immunogen and antigen (31). Gene sequences were assessed to identify potentially specific EBNA1 gene variability associated with those irregularities. Together with the previous reports from Serbian EBNA1 isolates, the dominance of P-Ala and P-Thr subtypes again confirmed one of the earliest hypotheses of European distribution in non-malignant diseases and geographically specific distribution (18) (55). On the other hand, EBNA1 variability patterns in this study did not at all correlate with previous findings in RA isolates (54). Interesting is that only P-Ala-sv-3 isolates lacked mucocutaneous presentation. This finding could be further investigated on a more significant number of isolates.

Although the LMP1 gene is significantly heterogeneous with greater variability than most other EBV genes, there is no literature on LMP1 polymorphisms in systemic autoimmune diseases. Even though this EBV oncogene is primarily significant in EBV-related cancers, its immunogenic potential, dependent on its variability, should be addressed. Thus, LMP1 variants with 30-bp deletion correlate with higher tumorigenic activity and lower immunogenic potential of EBV (15). One of those polymorphisms characterizes China1, identified in two of our isolates. According to some suggestions, China1-specific changes in Human leukocyte antigen (HLA) virus epitopes (within LMP1) could enable LMP1 expression, which leads to the inability to be recognized by LMP1-specific CD8+ cytotoxic T lymphocytes (56). It would be essential to follow whether patients with these isolates would develop some EBV-related cancers. With the identification of 4 different LMP1 variants, sequences from this study showed expected diversity for this geographic area. Due to the small sample and the diversity shown, specific LMP1 variants did not suggest any association with the clinical presentation of lupus. However, the largest group consisted of NC isolates, which should be investigated further because of the earlier hypothesis that the NC variant cannot inhibit T-cell proliferation and natural killer cytotoxicity due to amino acid substitutions responsible for immunosuppressive functions (56).

When it comes to study limitations, it would be more potent if the design was prospective with longitudinal follow-up of exposed and unexposed populations from the period without disease until the moment of SLE diagnosis. A follow-up period after the diagnosis of SLE would yield valuable results too. The same could be added to monitoring the influence of individual EBV gene polymorphisms on the potential development of the underlying disease and EBV-associated tumors. However, this kind of study would require large cohorts and would last long with an unpredictable outcome. There are more aspects related to other autoimmune diseases or other viruses and novel diagnostic tools that could be addressed also: lack of EBV sequences from other autoimmune diseases, serology data for other viruses, and droplet digital PCR for detection of low concentrations of EBV. Moreover, as immunological parameters were part of routine laboratory diagnosis and monitoring of SLE patients, these data for the study were collected from the patients’ medical records. Without additional immunological testing of the healthy participants, the analysis included immunological parameters only in the SLE population.

## 5 Conclusion

This study obtained a comprehensive analysis of serological, molecular and sequence markers of EBV infection in SLE patients, providing additional evidence and new hypotheses about the role of EBV infection in SLE development, particularly anti-EBV immune response. The results revealed even 24 times higher possibility of having SLE if there is the presence of anti-EBV-EA(D) IgG antibodies. Additionally, higher titers of anti-EBNA1 IgG antibodies and lower titers of anti-EBV-CA IgG antibodies were also found to be independently associated with the presence of lupus. Identification of independent viral factors associated with specific lupus manifestations for the first time revealed anti-EBV-EA(D) IgG antibodies as an independent factor associated with lymphopenia and a higher titer of anti-CA IgG antibodies as an independent factor associated with alopecia. Finally, the novel data on EBV EBNA1 and LMP1 gene polymorphisms in lupus have been reported.

## Data availability statement

The raw data supporting the conclusions of this article will be made available by the authors, without undue reservation.

## Ethics statement

The studies involving humans were approved by Ethical Board of the Faculty of Medicine, University of Belgrade (No 1550/IX-14). The studies were conducted in accordance with the local legislation and institutional requirements. The participants provided their written informed consent to participate in this study.

## Author contributions

AB: Conceptualization, Project administration, Supervision, Writing – original draft, Writing – review & editing. AC: Formal analysis, Methodology, Writing – review & editing. RM: Resources, Writing – review & editing. IJ: Methodology, Validation, Writing – review & editing. MG: Resources, Validation, Writing – original draft. MB: Investigation, Writing – original draft. IL: Data curation, Formal analysis, Writing – review & editing. SR: Resources, Writing – review & editing. AD: Data curation, Visualization, Writing – original draft. DM: Conceptualization, Investigation, Writing – review & editing.

## References

[1] Harley BJ Harley ITW Guthridge JM James JA. The curiously suspicious: A role for Epstein-Barr virus in lupus. Lupus (2006) 15(11):768–77. doi: 10.1177/0961203306070009 17153849

[2] Sternbæk L Draborg AH Østerlund MT Iversen LV Troelsen L Theander E. Increased antibody levels to stage-specific Epstein–Barr virus antigens in systemic autoimmune diseases reveal a common pathology. Scand J Clin Lab Invest. (2019) 79(1–2):7–16. doi: 10.1080/00365513.2018.1550807 30727744

[3] Balandraud N Roudier J. Epstein-Barr virus and rheumatoid arthritis. Jt Bone Spine. (2018) 85(2):165–70. doi: 10.1016/j.jbspin.2017.04.011 28499895

[4] Thorley-Lawson D. Epstein-Barr virus exploiting the immune system. Nat Rev Immunol (2001) 1(1):75–82. doi: 10.1038/35095584 11905817

[5] De Paschale M. Serological diagnosis of Epstein-Barr virus infection: Problems and solutions. World J Virol (2012) 1(1):31–43. doi: 10.5501/wjv.v1.i1.31 24175209 PMC3782265

[6] Gulley ML. Review molecular diagnosis of epstein-barr virus-related diseases. J Mol Diagn. (2001) 3(1):1–10. doi: 10.1016/S1525-1578(10)60642-3 11227065 PMC1907346

[7] Wood RA Guthridge L Thurmond E Guthridge CJ Kheir JM Bourn RL. Serologic markers of Epstein-Barr virus reactivation are associated with increased disease activity, inflammation, and interferon pathway activation in patients with systemic lupus erythematosus. J Transl Autoimmun (2021) 4:100117. doi: 10.1016/j.jtauto.2021.100117 35005588 PMC8716608

[8] Afrasiabi A Keane JT Ong LTC Alinejad-Rokny H Fewings NL Booth DR. Genetic and transcriptomic analyses support a switch to lytic phase in Epstein Barr virus infection as an important driver in developing Systemic Lupus Erythematosus. J Autoimmun (2022) 127:102781. doi: 10.1016/j.jaut.2021.102781 34952359

[9] Li ZX Zeng S Wu HX Zhou Y. The risk of systemic lupus erythematosus associated with Epstein–Barr virus infection: a systematic review and meta-analysis. Clin Exp Med (2019) 19(1):23–36. doi: 10.1007/s10238-018-0535-0 30361847 PMC6394567

[10] Draborg AH Lydolph MC Westergaard M Larsen SO Nielsen CT Duus K. Elevated concentrations of serum immunoglobulin free light chains in systemic lupus erythematosus patients in relation to disease activity, inflammatory status, B cell activity and Epstein-Barr virus antibodies. PloS One (2015) 10(9):e0138753. doi: 10.1371/journal.pone.0138753 26402865 PMC4581754

[11] Jog NR Young KA Munroe ME Harmon MT Guthridge JM Kelly JA. Association of Epstein-Barr virus serological reactivation with transitioning to systemic lupus erythematosus in at-risk individuals. Ann Rheum Dis (2019) 78(9):1235–41. doi: 10.1136/annrheumdis-2019-215361 PMC6692217 31217170

[12] Larsen M Sauce D Deback C Arnaud L Mathian A Miyara M. Exhausted cytotoxic control of epstein-barr virus in human lupus. PloS Pathog (2011) 7(10):e1002328. doi: 10.1371/journal.ppat.1002328 22028659 PMC3197610

[13] Kang I Quan T Nolasco H Park S-H Hong MS Crouch J. Defective control of latent epstein-barr virus infection in systemic lupus erythematosus. J Immunol (2004) 172(2):1287–94. doi: 10.4049/jimmunol.172.2.1287 14707107

[14] Draborg AH Jacobsen S Westergaard M Mortensen S Larsen JL Houen G. Reduced response to Epstein-Barr virus antigens by T-cells in systemic lupus erythematosus patients. Lupus Sci Med (2014) 1(1):e000015. doi: 10.1136/lupus-2014-000015 25396062 PMC4225738

[15] Knecht H Bachmann E Brousset P Sandvej K Nadal D Bachmann F. Deletions within the LMP1 oncogene of Epstein-Barr virus are clustered in Hodgkin’s disease and identical to those observed in nasopharyngeal carcinoma. Blood (1993) 82:2937–42. doi: 10.1182/blood.V82.10.2937.2937 8219183

[16] Ai J Xie Z Liu C Huang Z Xu J. Analysis of EBNA-1 and LMP-1 variants in diseases associated with EBV infection in Chinese children. Virol J (2012) 9:13. doi: 10.1186/1743-422X-9-13 22236445 PMC3269356

[17] Puchhammer-Stöckl E Görzer I. Cytomegalovirus and Epstein-Barr virus subtypes-The search for clinical significance. J Clin Virol (2006) 36(4):239–48. doi: 10.1016/j.jcv.2006.03.004 16697698

[18] Banko AV Lazarevic IB Karalic DZ Djukic VB Cupic MD Stevanovic G. The sequence analysis of Epstein–Barr virus EBNA1 gene: could viral screening markers for nasopharyngeal carcinoma be identified? Med Microbiol Immunol (2019) 208(1):81–8. doi: 10.1007/s00430-018-0561-2 30203133

[19] Tschochner M Leary S Cooper D Strautins K Chopra A Clark H. Identifying patient-specific Epstein-Barr Nuclear Antigen-1 genetic variation and potential autoreactive targets relevant to multiple sclerosis pathogenesis. PloS One (2016) 11(2):e0147567. doi: 10.1371/journal.pone.0147567 26849221 PMC4744032

[20] Hochberg M. Updating the American College of Rheumatology revised criteria for the classification of systemic lupus erythematosus. Arthritis Rheumatol (1997) 40(9):1725. doi: 10.1002/art.1780400928 9324032

[21] Matteo P Chessa E Morand EF Ugarte-gil MF Tektonidou M Van Vollenhoven R. Physician global assessment international standardisation COnsensus in systemic lupus erythematosus: the PISCOS. Lancet Rheumatol (2022) 4(6):e441–9. doi: 10.1016/S2665-9913(22)00107-2 38293958

[22] Holloway L Humphrey L Heron L Pilling C Kitchen H Højbjerre L. Patient-reported outcome measures for systemic lupus erythematosus clinical trials: A review of content validity, face validity and psychometric performance. Health Qual Life Outcomes. (2014) 12:116. doi: 10.1186/s12955-014-0116-1 25048687 PMC4223409

[23] Tselios K Gladman DD Urowitz MB. How can we define low disease activity in systemic lupus erythematosus? Semin Arthritis Rheum (2019) 48(6):1035–40. doi: 10.1016/j.semarthrit.2018.10.013 30415943

[24] Zen M Iaccarino L Gatto M Saccon F Larosa M Ghirardello A. Lupus low disease activity state is associated with a decrease in damage progression in Caucasian patients with SLE, but overlaps with remission. Ann Rheum Dis (2018) 77(1):104–10. doi: 10.1136/annrheumdis-2017-211613 28970217

[25] Banko AV Lazarevic IB Folic MM Djukic VB Cirkovic AM Karalic DZ. Characterization of the variability of Epstein-Barr virus genes in nasopharyngeal biopsies: Potential predictors for carcinoma progression. PloS One (2016) 11(4):0153498. doi: 10.1371/journal.pone.0153498 PMC4829223 27071030

[26] Lorenzetti MA Altcheh J Moroni S Moscatelli G Chabay PA Preciado MV. EBNA1 sequences in Argentinean pediatric acute and latent epstein-barr virus infection reflect circulation of novel South American variants. J Med Virol (2010) 82(10):1730–8. doi: 10.1002/jmv.21871 20827771

[27] Li DJ Bei JX Mai SJ Xu JF Chen LZ Zhang RH. The dominance of China 1 in the spectrum of Epstein-Barr virus strains from cantonese patients with nasopharyngeal carcinoma. J Med Virol (2009) 81(7):1253–60. doi: 10.1002/jmv.21503 19475622

[28] Hall T. BioEdit: A user-friendly biological sequence alignment editor and analysis program for Windows 95, 98, NT, 2000, XP. Nucl Acids Symp (1999) 41:95–8.

[29] Edwards RH Seillier-Moiseiwitsch F Raab-Traub N. Signature amino acid changes in latent membrane protein 1 distinguish epstein-barr virus strains. Virology (1999) 261:79–95. doi: 10.1006/viro.1999.9855 10441557

[30] Jog NR James JA. Epstein barr virus and autoimmune responses in systemic lupus erythematosus. Front Immunol (2021) 11:623944. doi: 10.3389/fimmu.2020.623944 33613559 PMC7886683

[31] Laurynenka V Ding L Kaufman KM James JA Harley JB. A high prevalence of anti-EBNA1 heteroantibodies in systemic lupus erythematosus (SLE) supports anti-EBNA1 as an origin for SLE autoantibodies. Front Immunol (2022) 13:830993. doi: 10.3389/fimmu.2022.830993 35251022 PMC8892314

[32] Miljanovic D Cirkovic A Jermic I Basaric M Lazarevic I Grk M. Markers of epstein–barr virus infection in association with the onset and poor control of rheumatoid arthritis: A prospective cohort study. Microorganisms (2023) 11(8):1958. doi: 10.3390/microorganisms11081958 37630516 PMC10459700

[33] McClain MT Poole BD Bruner BF Kaufman KM Harley JB James JA. An altered immune response to Epstein-Barr nuclear antigen 1 in pediatric systemic lupus erythematosus. Arthritis Rheumatol (2006) 54(1):360–8. doi: 10.1002/art.21682 16385527

[34] Vista ES Weisman MH Ishimori ML Chen H Bourn RL Bruner BF. Strong viral associations with SLE among Filipinos. Lupus Sci Med (2017) 4(1):e000214. doi: 10.1136/lupus-2017-000214 29214036 PMC5704743

[35] Chougule D Nadkar M Rajadhyaksha A Pandit-Shende P Surve P Dawkar N. Association of clinical and serological parameters of systemic lupus erythematosus patients with Epstein-Barr virus antibody profile. J Med Virol (2018) 90(3):559–63. doi: 10.1002/jmv.24904 28734074

[36] Parks CG Cooper GS Hudson LL Dooley MA Treadwell EL St.Clair EW. Association of Epstein-Barr virus with systemic lupus erythematosus: Effect modification by race, age, and cytotoxic T lymphocyte-associated antigen 4 genotype. Arthritis Rheumatol (2005) 52(4):1148–59. doi: 10.1002/art.20997 15818712

[37] Bjornevik K Cortese M Healy BC Kuhle J Mina MJ Leng Y. Longitudinal analysis reveals high prevalence of Epstein-Barr virus associated with multiple sclerosis. Sci (80- ). (2022) 375(6578):296–301. doi: 10.1126/science.abj8222 35025605

[38] Quaglia M Merlotti G De Andrea M Borgogna C Cantaluppi V. Review viral infections and systemic lupus erythematosus: New players in an old story. Viruses (2021) 13(2):277. doi: 10.3390/v13020277 33670195 PMC7916951

[39] Mok CC. Current role of rituximab in systemic lupus erythematosus. Int J Rheum Dis (2015) 18(2):154–63. doi: 10.1111/1756-185X.12463 25522652

[40] Draborg A Izarzugaza JMG Houen G. How compelling are the data for Epstein-Barr virus being a trigger for systemic lupus and other autoimmune diseases? Curr Opin Rheumatol (2016) 28(4):398–404. doi: 10.1097/BOR.0000000000000289 26986247

[41] França SA de S Viana JBG de O Góes HCA Fonseca RR de S Laurentino RV Costa IB. Epidemiology of the epstein–barr virus in autoimmune inflammatory rheumatic diseases in northern Brazil. Viruses (2022) 14(4):694. doi: 10.3390/v14040694 35458425 PMC9028150

[42] Gross AJ Hochberg D Rand WM Thorley-Lawson DA. EBV and systemic lupus erythematosus: A new perspective. J Immunol (2005) 174(11):6599–607. doi: 10.4049/jimmunol.174.11.6599 15905498

[43] Trier NH Holm BE Heiden J Slot O Locht H Lindegaard H. Antibodies to a strain-specific citrullinated Epstein-Barr virus peptide diagnoses rheumatoid arthritis. Sci Rep (2018) 8(1):3684. doi: 10.1038/s41598-018-22058-6 29487382 PMC5829227

[44] Draborg AH Jørgensen JM Müller H Nielsen CT Jacobsen S Iversen LV. Epstein-Barr virus early antigen diffuse (EBV-EA/D)-directed immunoglobulin A antibodies in systemic lupus erythematosus patients. Scand J Rheumatol (2012) 41(4):280–9. doi: 10.3109/03009742.2012.665944 22646970

[45] Ramos-Casals M Sanz I Bosch X Stone JH Khamashta MA. B-cell-depleting therapy in systemic lupus erythematosus. Am J Med (2012) 125(4):327–36. doi: 10.1016/j.amjmed.2011.09.010 PMC3925418 22444096

[46] Han L Zhang Y Wang Q Xin M Yang K Lei K. Epstein–Barr virus infection and type I interferon signature in patients with systemic lupus erythematosus. Lupus (2018) 27(6):947–54. doi: 10.1177/0961203317753069 29338588

[47] Truszewska A Wirkowska A Gala K Truszewski P Krzemień-Ojak Ł Mucha K. EBV load is associated with cfDNA fragmentation and renal damage in SLE patients. Lupus (2021) 30(8):1214–25. doi: 10.1177/09612033211010339 33866897

[48] Piroozmand A Kashani HH Zamani B. Correlation between Epstein-Barr virus infection and disease activity of systemic lupus erythematosus: A cross-sectional study. Asian Pacific J Cancer Prev (2017) 18(2):523–7. doi: 10.22034/APJCP.2017.18.2.523 PMC5454753 28345840

[49] Yu XX Yao CW Tao JL Yang C Luo MN Li SM. The expression of renal Epstein-Barr virus markers in patients with lupus nephritis. Exp Ther Med (2014) 7(5):1135–40. doi: 10.3892/etm.2014.1578 PMC3991543 24940399

[50] Bjørnevik K Riise T Bostrom I Casetta I Cortese M Granieri E. Negative interaction between smoking and EBV in the risk of multiple sclerosis: The EnvIMS study. Mult Scler. (2017) 23(7):1018–24. doi: 10.1177/1352458516671028 27663872

[51] Katz BZ Salimi B Kim S Nsiah-Kumi P And BA Wagner-Weiner L. Epstein-Barr virus burden in adolescents with systemic lupus erythematosus. Pediatr Infect Dis J (2001) 20(2):148–53. doi: 10.1097/00006454-200102000-00006 11224832

[52] Gulley ML Tang W. Laboratory assays for Epstein-Barr virus-related disease. J Mol Diagnostics. (2008) 10(4):279–92. doi: 10.2353/jmoldx.2008.080023 PMC2438195 18556771

[53] Mechelli R Manzari C Policano C Annese A Picardi E Umeton R. Epstein-Barr virus genetic variants are associated with multiple sclerosis. Neurology (2015) 84(13):1362–8. doi: 10.1212/WNL.0000000000001420 PMC4388746 25740864

[54] Masuoka S Kusunoki N Takamatsu R Takahashi H Tsuchiya K Kawai S. Epstein-Barr virus infection and variants of Epstein-Barr nuclear antigen-1 in synovial tissues of rheumatoid arthritis. PloS One (2018) 13(12):e0208957. doi: 10.1371/journal.pone.0208957 30533036 PMC6289453

[55] Gutiérrez M Raj A Spangler G Sharma A Hussain A Judde J. Sequence variations in EBNA-1 may dictate restriction of tissue distribution of Epstein-Barr virus in normal and tumour cells. J Gen Virol (1997) 78:1663–70. doi: 10.1099/0022-1317-78-7-1663 9225043

[56] Edwards RH Sitki-Green D Moore DT Raab-Traub N. Potential selection of LMP1 variants in nasopharyngeal carcinoma. J Virol (2004) 78(2):868–81. doi: 10.1128/JVI.78.2.868-881.2004 PMC368819 14694118

