# Supplementary material for: Epstein-Barr virus infection as potential indicator of the occurrence and clinical presentation of systemic lupus erythematosus
Source: Front Immunol. 2023 Dec 7;14:1307589. doi: 10.3389/fimmu.2023.1307589 (PMC10749334; doi:10.3389/fimmu.2023.1307589)
Supplement: Supplementary file 1 [file Table_1.docx]

Supplementary material

Table 1. Therapeutically approaches in active and latent EBV infection within SLE patients

| **Medication, n (%)** | **Active EBV infection** | **Latent EBV infection** | **p^*^** |
| --- | --- | --- | --- |
| Immunosuppressive drugs | 8 (18.6) | 22 (36.7) | **0.047** |
| Corticosteroids | 38 (88.4) | 52 (86.7) | 0.797 |
| Antimalarials | 34 (79.1) | 52 (86.7) | 0.306 |
| Pulse therapy | 5 (11.6) | 8 (13.6) | 0.773 |

^*^for the level of significance of 0.05, according to chi-square test

Table 2. Regression modeling – univariate analysis for all outcomes (just p values are reported)

| Parameter | Outcome | | | | | | | | | |
| --- | --- | --- | --- | --- | --- | --- | --- | --- | --- | --- |
|  | SLE | Active SLE | Rash | Arthritis | Mucosal ulcerations | Alopecia | Lupus nephritis | Leucopenia | Lymphopenia | Thrombocytopenia |
| Age | **<0.001** | **0.002** | 0.058 | **0.036** | 0.403 | 0.820 | **0.042** | 0.592 | **0.008** | 0.077 |
| Gender | 0.433 | 0.679 | 0.190 | 0.513 | 0.999 | 0.273 | 0.412 | 0.821 | 1.000 | 0.974 |
| Smoker | **0.004** | 0.558 | 0.791 | 0.860 | 0.794 | 0.677 | 0.281 | 0.814 | 0.774 | 0.967 |
| Smoking duration | **<0.001** | 0.285 | 0.770 | 0.611 | 0.971 | 0.490 | 0.327 | 0.057 | **0.016** | **0.036** |
| BMI | 0.076 | 0.635 | 0.493 | 0.377 | 0.186 | 0.735 | 0.491 | 0.229 | 0.051 | 0.094 |
| Hashimoto thyroiditis | 0.769 | 0.856 | 0.849 | 0.849 | **0.020** | 0.368 | 0.598 | 0.608 | 0.453 | 0.079 |
| HTA | 0.497 | 0.834 | 0.083 | 0.640 | 0.938 | **0.027** | 0.385 | 0.096 | 0.109 | 0.270 |
| DM | 0.286 | 0.533 | 0.529 | 0.839 | 0.170 | 0.864 | 0.601 | 0.999 | 0.696 | 0.999 |
| Cardiovascular events | 0.078 | 0.679 | 0.190 | 0.931 | 0.958 | 0.100 | 0.999 | 0.821 | 0.727 | 0.999 |
| Number of ACR criteria | NA | **<0.001** | NA | **0.012** | **0.001** | 0.181 | **0.009** | NA | **0.004** | NA |
| ESR | **0.013** | **<0.001** | 0.061 | **0.001** | 0.773 | 0.085 | 0.277 | 0.795 | **0.005** | 0.593 |
| CRP | 0.054 | **0.022** | 0.596 | **0.040** | 0.810 | 0.105 | 0.443 | 0.763 | 0.073 | 0.459 |
| NLR | 0.604 | 0.268 | 0.198 | **0.038** | 0.602 | 0.313 | 0.346 | 0.083 | **<0.001** | 0.380 |
| EBV DNA | 0.893 | 0.099 | 0.745 | 0.319 | 0.480 | 0.805 | 0.246 | 0.640 | 0.063 | 0.848 |
| Anti-EBV-EBNA1 IgG titer | **<0.001** | **0.003** | 0.599 | 0.146 | 0.276 | 0.464 | 0.619 | 0.720 | 0.242 | 0.878 |
| Anti-EBV-CA IgG titer | **0.043** | 0.932 | 0.169 | 0.794 | 0.248 | **0.011** | 0.422 | 0.350 | 0.549 | 0.142 |
| Anti-EBV-CA IgM titer | **0.001** | 0.240 | 0.196 | 0.171 | 0.180 | 0.320 | 0.053 | 0.408 | 0.873 | **0.046** |
| Anti-EBV-EA(D) IgG titer | **<0.001** | 0.322 | 0.995 | 0.363 | 0.553 | 0.647 | 0.144 | 0.561 | **0.011** | 0.514 |
| Anti-EBV-EA(D) IgM titer | 0.055 | 0.807 | 0.810 | 0.512 | 0.381 | 0.176 | 0.368 | 0.227 | **0.023** | 0.485 |
| EBV infection status (active) | **<0.001** | 0.241 | 0.924 | 0.601 | 0.223 | 0.649 | 0.444 | 0.928 | 0.839 | 0.815 |
| Anti-EBV-EBNA1 IgG presence | 0.443 | 0.545 | 0.759 | 0.759 | 0.999 | 0.999 | 0.896 | 0.507 | 0.565 | 0.371 |
| Anti-EBV-CA IgG presence | 1.000 | NA | NA | NA | NA | NA | NA | NA | NA | NA |
| Anti-EBV-CA IgM presence | **<0.001** | 0.222 | 0.078 | 0.197 | 0.173 | 0.445 | 0.054 | 0.758 | 0.817 | 0.386 |
| Anti-EBV-EA(D) IgG presence | **<0.001** | 0.650 | 0.589 | 0.427 | 0.960 | 0.650 | 0.116 | 0.883 | 0.207 | 0.247 |
| Anti-EBV-EA(D) IgM presence | **0.010** | 0.905 | 0.590 | 0.624 | 0.289 | 0.181 | 0.599 | 0.193 | 0.051 | 0.628 |
| ANA, med (min-max) | NA | **0.002** | **0.005** | **0.001** | 0.158 | **0.030** | 0.452 | 0.157 | **<0.001** | 0.346 |
| Anti-dsDNA positivity, n (%) | NA | **0.038** | 0.251 | **0.035** | 0.387 | 0.792 | **0.003** | 0.859 | **0.020** | 0.258 |
| Anti-SSA positivity, n (%) | NA | 0.502 | 0.253 | 0.965 | 0.625 | 0.058 | 0.156 | **0.028** | 0.411 | 0.847 |
| Anti-Sm positivity, n (%) | NA | **0.001** | **0.016** | **0.001** | 0.308 | **0.050** | 0.995 | 0.128 | **0.010** | 0.232 |
| aCL-IgM positivity, n (%) | NA | 0.665 | 0.816 | 0.126 | 0.553 | 0.053 | 0.880 | 0.437 | 0.335 | 0.614 |
| aCL-IgG positivity, n (%) | NA | 0.629 | 0.573 | 0.976 | 0.499 | 1.000 | 0.098 | 0.390 | 0.707 | 0.558 |
| Anti- β2-GPI IgG positivity, n (%) | NA | 0.967 | 0.767 | 0.767 | 0.891 | 0.168 | **0.050** | 0.972 | 0.181 | 0.999 |
| Anti-β2-GPI IgM positivity, n (%) | NA | 0.457 | 0.284 | 0.999 | 0.891 | 0.920 | 0.245 | 0.972 | 0.365 | 0.825 |
| RF positivity, n (%) | NA | **0.037** | **0.017** | **0.017** | 0.234 | **0.008** | 0.880 | 0.314 | 0.168 | 0.117 |
| C3, mean±sd | NA | **0.002** | **0.005** | **0.025** | 0.245 | 0.153 | **<0.001** | 0.598 | **0.003** | 0.644 |
| C4, med (min-max) | NA | 0.066 | 0.079 | 0.272 | 0.417 | 0.094 | 0.235 | 0.161 | 0.283 | 0.577 |
| Total IgG, mean±sd | NA | **0.019** | 0.184 | **0.005** | 0.539 | 0.373 | 0.064 | 0.091 | **0.012** | 0.791 |
| Immunosupressive therapy |  | 0.195 | 0.228 | 0.455 | 0.323 | 0.085 | 0.162 | 0.996 | 0.911 | 0.225 |

Table 3. Factors associated with Systemic Lupus Erythematosus

| **Factor** | **Univariate logistic regression** | | |
| --- | --- | --- | --- |
|  | **OR** | **95% CI OR** | **p^*^** |
| Age | 0.946 | 0.92-0.97 | <0.001 |
| Smoker | 2.308 | 1.31-4.07 | 0.004 |
| Smoking duration | 1.053 | 1.02-1.08 | <0.001 |
| ESR | 1.028 | 1.01-1.05 | 0.013 |
| Anti-EBV-EBNA1 IgG titer | 1.008 | 1.00-1.01 | <0.001 |
| Anti-EBV-CA IgG titer | 0.995 | 0.99-1.00 | 0.043 |
| Anti-EBV-CA IgM presence | 10.805 | 3.15-37.05 | <0.001 |
| Anti-EBV-CA IgM titer | 3,914 | 1.79-8.57 | 0.001 |
| Anti-EBV-EA(D) IgG presence | 9.676 | 3,60-25.98 | <0.001 |
| Anti-EBV-EA(D) IgG titer | 1.030 | 1.01-1.05 | <0.001 |
| Anti-EBV-EA(D) IgM presence | 3.090 | 1.30-7.32 | 0.010 |
| Active EBV infection | 7.087 | 3.22-15.61 | <0.001 |

*for the level of significance of 0.05

Table 4. Factors associated with the active form of SLE

| **Factor** | **Univariate logistic regression** | | |
| --- | --- | --- | --- |
|  | **OR** | **95% CI OR** | **p^*^** |
| Age | 0,949 | 0,92-0,98 | 0.002 |
| Number of positive ACR criteria | 1.939 | 1.34-2.80 | <0.001 |
| ESR | 1.043 | 1.02-1.07 | <0.001 |
| CRP | 1.061 | 1.01-1.12 | 0.022 |
| ANA Ab titer | 1.003 | 1.01-1.01 | 0.002 |
| Anti-dsDNA presence | 2.696 | 1.06-6.87 | 0.038 |
| Anti-Sm positivity | 5.760 | 2.01-16.48 | 0.001 |
| C3 concentration | 0.065 | 0.01-0.36 | 0.002 |
| Total serum IgG level | 1.106 | 1.02-1.20 | 0.019 |
| Positive RF | 3.048 | 1.07-8.66 | 0.037 |
| Anti-EBV-EBNA1 IgG Ab titer | 1.006 | 1.01-1.01 | 0.003 |

*for the level of significance of 0.05

Table 5. Factors associated with rash

| **Factor** | **Univariate logistic regression** | | |
| --- | --- | --- | --- |
|  | **OR** | **95% CI OR** | **p^*^** |
| ANA titer | 1.003 | 1.01-1.01 | 0.005 |
| C3 level | 0.084 | 0.02-0.47 | 0.005 |
| Positive RF | 3.536 | 1.25-10.01 | 0.017 |
| Anti-Sm presence | 3.314 | 1.25-8.80 | 0.016 |
|  | Step 2 – Multivariate logistic regression, adjusted for age | | |
| C3 | 0.049 | 0.01-0.44 | **0.007** |
| Positive RF | 3.363 | 1.10-10.31 | **0.034** |

*for the level of significance of 0.05

Table 6. Factors associated with arthritis

| **Factor** | **Univariate logistic regression** | | |
| --- | --- | --- | --- |
|  | **OR** | **95% CI OR** | **p^*^** |
| Age | 0.965 | 0.93-0.99 | 0.036 |
| Number of positive ACR criteria | 1.471 | 1.09-1.99 | 0.012 |
| ESR | 1.034 | 1.01-1.05 | 0.001 |
| CRP | 1.035 | 1.01-1.07 | 0.040 |
| NLR | 1.218 | 1.01-1.47 | 0.038 |
| ANA titer | 1.004 | 1.00-1.01 | 0.001 |
| Anti-dsDNA presence | 2.762 | 1.08-7.09 | 0.035 |
| C3 level | 0.153 | 0.03-0.79 | 0.025 |
| Total serum IgG level | 1.136 | 1.04-1.24 | 0.005 |
| Positive RF | 3.536 | 1.25-10.01 | 0.017 |
| Anti-Sm presence | 5.526 | 2.01-15.18 | 0.001 |

*for the level of significance of 0.05

Table 7. Factors associated with mucosal ulcerations

| **Factor** | **Univariate logistic regression** | | |
| --- | --- | --- | --- |
|  | **OR** | **95% CI OR** | **p^*^** |
| Hashimoto thyroiditis | 4.643 | 1.27-17.01 | 0.020 |
| Number of positive ACR criteria | 1.997 | 1.32-3002 | 0.001 |
| Secondary Sjogren syndrome | 3.871 | 1.08-13.87 | 0.038 |
|  | Step 2 – Multivariate logistic regression, adjusted for age | | |
| Number of positive ACR criteria | 2.153 | 1.38-3.37 | 0.001 |
| Secondary Sjogren syndrome | 5.857 | 1.18-29.08 | 0.031 |

*for the level of significance of 0.05

Table 8. Factors associated with alopecia

| **Factor** | **Univariate logistic regression** | | |
| --- | --- | --- | --- |
|  | **OR** | **95% CI OR** | **p^*^** |
| ANA titer | 1.002 | 1.00-1.01 | 0.030 |
| HTA | 2.495 | 1.11-5.61 | 0.027 |
| RF positive | 4.333 | 1.47-12.76 | 0.008 |
| Anti-Sm presence | 2.631 | 1.00-6.92 | 0.050 |
| Anti-EBV-CA IgG titer | 1.020 | 1.01-1.04 | 0.018 |
|  | Step 2 – Multivariate logistic regression, adjusted for age | | |
| RF positive | 4.871 | 1.52-15.61 | 0.008 |
| Anti-EBV-CA IgG titer | 1.015 | 1.01-1.03 | 0.019 |

*for the level of significance of 0.05

Table 9. Factors associated with lupus nephritis

| **Factor** | **Univariate logistic regression** | | |
| --- | --- | --- | --- |
|  | **OR** | **95% CI OR** | **p^*^** |
| Age | 0.961 | 0.93-0.99 | 0.042 |
| Number of positive ACR criteria | 1.533 | 1.12-2.11 | 0.009 |
| Anti-dsDNA presence | 4.724 | 1.72-12.98 | 0.003 |
| C3 | 0.016 | 0.01-1.14 | <0.001 |
| Antiβ2 IgG presence | 4.842 | 1.00-23.53 | 0.050 |

*for the level of significance of 0.05

Table 10. Factors associated with leucopenia

| **Factor** | **Univariate logistic regression** | | |
| --- | --- | --- | --- |
|  | **OR** | **95% CI OR** | **p^*^** |
| anti-SSA presence | 4.676 | 1.18-18.51 | 0.028 |
|  | Multivariate logistic regression, adjusted for age | | |
| anti-SSA presence | 4.763 | 1.20-18.94 | 0.027 |

*for the level of significance of 0.05

Table 11. Factors associated with lymphopenia

| **Factor** | **Univariate logistic regression** | | |
| --- | --- | --- | --- |
|  | **OR** | **95% CI OR** | **p^*^** |
| Age | 0.957 | 0.93-0.99 | 0.008 |
| Smoking duration | 0.939 | 0.89-0.99 | 0.016 |
| ESR | 1.032 | 1.01-1.06 | 0.005 |
| NLR | 2.517 | 1.57-4.03 | <0.001 |
| ANAtiter | 1.004 | 1.00-1.01 | <0.001 |
| Anti-dsDNApresence | 3.451 | 1.22-9.76 | 0.020 |
| C3 level | 0.082 | 0.02-0.44 | 0.003 |
| Total serum IgG level | 1.119 | 1.03-1.22 | 0.012 |
| Anti-Sm presence | 4.267 | 1.42-12.83 | 0.010 |
| Anti-EBV-EA(D) IgG Abs titer | 1.012 | 1.01-1.02 | 0.011 |
| Anti-EBV-EA(D) IgM Abs titer | 0.537 | 0.31-0.92 | 0.023 |
| No of ACR criteria | 1.665 | 1.18-2.35 | 0.004 |

*for the level of significance of 0.05

Table 12. Factors associated with thrombocytopenia

| **Factor** | **Univariate logistic regression** | | |
| --- | --- | --- | --- |
|  | **OR** | **95% CI OR** | **p^*^** |
| Smoking duration | 0.854 | 0.74-0.99 | 0.036 |
| Anti-EBV-CA IgM Abs titer | 1.653 | 1.01-2.71 | 0.046 |
|  | Multivariate logistic regression, adjusted for age | | |
| Smoking duration | 0.854 | 0.74-0.99 | 0.036 |
